# Preliminary study of noninvasive prenatal screening for 22q11.2 deletion/duplication syndrome using multiplex dPCR assay

**DOI:** 10.1186/s13023-023-02903-2

**Published:** 2023-09-08

**Authors:** Jing Wang, Wei Wang, Wenbo Zhou, Yan Zhou, Linna Zhou, Xinyue Wang, Bin Yu, Bin Zhang

**Affiliations:** 1 Changzhou Maternity and Child Health Care Hospital, Changzhou, 213003 Jiangsu Province China; 2 Xingzhi Biotechnology Co., LTD, Suzhou, 215000 Jiangsu Province China

**Keywords:** 22q11.2 deletion/duplication syndrome, Noninvasive prenatal screening, Digital PCR (dPCR)

## Abstract

**Objective:**

This study aimed to establish a cell-free fetal DNA (cffDNA) assay using multiplex digital PCR (dPCR) for identifying fetuses at increased risk of 22q11.2 deletion/duplication syndrome.

**Methods:**

Six detection sites and their corresponding probes were designed for the 22q11.2 recurrent region. A dPCR assay for the noninvasive screening of 22q11.2 deletion/duplication syndrome was established. A total of 130 plasma samples from pregnant women (including 15 samples with fetal 22q11.2 deletion/duplication syndrome) were blindly tested for evaluating the sensitivity and specificity of the established assay.

**Results:**

DNA with different sizes of 22q11.2 deletion/duplication was detected via dPCR, indicating that the designed probes and detection sites were reasonable and effective. In the retrospective clinical samples, 11 out of 15 samples of pregnant women with 22q11.2 deletion/duplication were detected during the cffDNA assay, and accurate regional localization was achieved. Among the 115 normal samples, 111 were confirmed to be normal. Receiver operating characteristic curves were used for assessing the cut-off values and AUC for these samples. The sensitivity, specificity, and positive as well as negative predictive values were 73.3%, 96.5%, 73.3%, and 96.5%, respectively.

**Conclusion:**

The cffDNA assay based on dPCR technology for the noninvasive detection of 22q11.2 recurrent copy number variants in fetuses detected most affected cases, including smaller but relatively common nested deletions, with a low false-positive rate. It is a potential, efficient and simple method for the noninvasive screening of 22q11.2 deletion/duplication syndrome.

**Supplementary Information:**

The online version contains supplementary material available at 10.1186/s13023-023-02903-2.

## Background

Copy number variants (CNVs) are ubiquitous in the human genome. Some CNVs exist in the normal human population in the form of genetic polymorphisms, whereas others may be related to human traits and diseases. CNVs occur frequently in certain regions, known as the recurrent regions, because of non-allelic homologous recombination, end-joining replication fork stagnation, and fork stalling and template switching [1]. The occurrence frequency of the 22q11.2 region is approximately 1/4000 in the human population and as high as 1/800–1/1000 in fetuses. Duplications and deletions are both equally common. The 22q11.2 deletion/duplication syndrome is the second most common genetic cause for developmental delay and congenital heart disease following Down syndrome, and more commonly occurring than trisomy 18 and trisomy 13 combined [2, 3]. The 22q11.2 deletion syndrome present heterogeneously including multiple congenital anomalies and later-onset conditions, such as congenital heart disease (tetralogy of Fallot, aortic arch malformation, ventricular septal defect, etc.), palatal malformation (cleft palate and velopharyngeal insufficiency), and characteristic facial deformity [4, 5]. Antenatal diagnosis or screening of 22q11.2 deletion syndrome ensures timely treatment for neonatal hypocalcemia and immunodeficiency, improving treatment outcomes [6, 7]. The 22q11.2 duplication syndrome varies widely in penetrance and presentation, with some patients having mild to severe abnormalities, usually including facial deformities, cardiac abnormalities, autism spectrum disorders (ASD), and neurological deficits (OMIM # 608,363) [8, 9]. The incidence of ASD in 22q11.2 duplication syndrome is 14–25%, which is the highest among all genetic diseases. It is suggested that prospective medical screening should be performed in all patients with 22q11.2 duplication syndrome [3, 10].

Screening for 22q11.2 deletion/duplication syndrome in fetuses is difficult because of the lack of effective targeted programs. Currently, screening is performed primarily through fetal ultrasound [11]. Ultrasound detects fetal abnormalities and is followed by diagnostic testing, thus detecting 22q11.2 deletion/duplication syndrome on a microarray. Due to many factors, such as the individual fetal development, standard of ultrasound technology and operators, most abnormal fetuses are not detected until the second or third trimester [12]. In addition, 22q11.2 deletion/duplication syndrome is easily missed in fetuses because of the high heterogeneity in its presentation [13].

Discovery of circulating cell-free fetal DNA (cffDNA) in the maternal plasma has enabled the development of noninvasive prenatal screening and diagnostic techniques. In recent years, noninvasive prenatal screening based on next-generation sequencing (NGS) technology has been mainly used for detecting common fetal aneuploidy with high sensitivity and specificity; however its use is relatively low for fetal CNVs detection [14–16]. Studies for improving the technology have been conducted. These include increasing the sequencing depth, adding probes to custom chips, changing the algorithm to enhance detection effect accuracy, such as single nucleotide polymorphism (SNP) based digital analysis of selected areas (DANSR) and targeted capture enrichment assay (TCEA) technology [17]. However, due to the significant increase in detection costs, promoting the clinical application of these methods is difficult.

Studies have reported that digital PCR (dPCR) technology has a positive effect on fetal T21/T18 noninvasive prenatal screening [18, 19]. dPCR is a nucleic acid absolute quantification technology that can be used for absolute quantification of the initial sample [20]. In this process, the PCR reaction is randomly distributed to tens of thousands of independent partitions. After amplification, PCR partitions are read and counted as negative or positive based on their fluorescence amplitude, in which minute differences in the abundance of target sequences can be accurately detected. Finally, Poisson distribution is used to calculate the number of target sequences. This technique has high sensitivity, accuracy, stability, and tolerance [20, 21]. dPCR is sufficiently precise for detecting fetal chromosomal aneuploidy in maternal plasma and suitable for noninvasive prenatal diagnosis of certain monogenic diseases, such as hemophilia, fetal sex detection, rhesus blood group D antigen genotyping, and achondroplasia [22–26].

In this study, we developed a dPCR assay for noninvasive detection of the 22q11.2 recurrent CNV in fetuses, and preliminarily evaluated its effectiveness through retrospective research.

## Materials and methods

### Ethics approval and consent for participation

The design and protocol of this study were reviewed and approved by the ethics committee of Changzhou Maternity and Child Health Care Hospital (No. 2022014). All pregnant women received genetic counseling and provided informed consent prior to testing.

### Clinical subjects

This study recruited patients who had undergone chromosomal microarray (CMA) testing from the Prenatal Diagnosis Center of Changzhou Maternal and Child Health Hospital.

1. Subjects for the preliminary evaluation of probe effectiveness: Four patients with differently sized 22q11.2 deletion/duplication identified via CMA were recruited for this study.

2. Subjects for the retrospective evaluation: A. True positive group: 15 pregnant women with fetal CNV at 22q11.2, aged 21–36 years, gestational age 18 + 3 w–26 w. B: Negative group: 115 pregnant women with normal fetuses, aged 20–46 years, gestational age 17 + 1 w–27 w.

Maternal blood samples were collected prior to amniocentesis. Plasma was isolated from blood samples, stored at -80 °C, and retrospectively analyzed. The samples were then blind-coded and processed by operators and analysts.

### Sample collection and DNA extraction

#### Genomic DNA extraction

Blood samples (2 ml) were collected in EDTA tubes, and genomic DNA was extracted using the Puregene Blood Core Kit C (Qiagen, Hilden, Germany) according to the manufacturer’s instructions. Amniotic fluid (10 ml) was obtained from each pregnant woman via amniocentesis. DNA was extracted from the amniotic fluid using a QIAamp DNA Mini Kit (Qiagen Inc., Valencia, CA, USA) following the manufacturer’s instructions.

#### Plasma separation and cffDNA extraction

Peripheral blood (10 ml) was collected from pregnant women via simple needle aspiration and centrifuged at 1600 g at 4 °C for 10 min and again at 16,000 g for 10 min to obtain cell-free plasma. Cell-free DNA was extracted from 2 mL plasma using a Magbead Free-Circulating DNA Maxi Kit (KangWei Shiji, China), according to the manufacturer’s instructions. A magnetic bead-based cell-free fetal DNA (cffDNA) enrichment kit (Magnetic Bead-Based cffDNA Purification Kit) (Xingzhi Biotechnology, China) was used for enrichment to enhance the proportion of cffDNA, following the manufacturer’s instructions.

#### CMA

DNA (250 ng) was amplified, labeled, and hybridized to a GCS 3000Dx v.2 microarray platform (Affymetrix, CA, USA). The SNP array test was performed using a commercial 750 K microarray chip (Affymetrix CytoScan 750 K Array). After hybridization with fragmented DNA, the chip was washed with buffer and scanned using a GeneChip Scanner 3000 7G. Data was analyzed using the Chromosome Analysis Suite v4.1 (ChAs) software package. Public databases (DECIPHER, OMIM, ClinVar, ISCA, NCBI, and UCSC) were used to retrieve the data. The pathogenicity of the identified CNVs was evaluated according to the American College of Medical Genetics and Genomics (ACMG) guidelines.

#### Target specific primer and probe designs

**Fig. 1:**
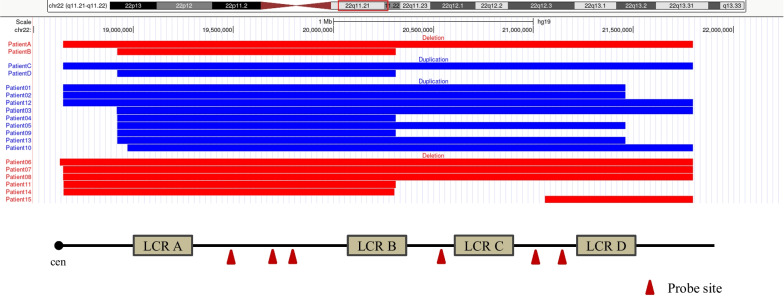
Depiction of the 22q11.2 region in chromosome 22 and the probe site. The ideogram of chromosome 22 and the 22q11.2 highlighted in a small red box are shown on top. This region includes four sets of LCR referred to as LCR-A, LCR-B, LCR-C, and LCR-D (brown boxes). The probes sites of dPCR are marked in red. Schematic of the CMA results of the four patients in the preliminary assessment of probe effectiveness study and 15 patients in retrospective study. The red bars represent deletion sizes, and blue bars represent duplication sizes

The 22q11.2 recurrent region contains four hot spot regions known as A–D low-copy repeats (LCR) on the long arm of the chromosome 22 [27]. Three sequence tagged sites in the proximal segment (LCR A-B) and central segment (LCR B-D) were selected as detection targets (Fig. 1). Two housekeeping genes on chromosome 1 were selected as the reference gene testing loci.

Candidate primers and probes were chosen using the online nucleotide BLAST and primer-BLAST tools provided by the National Center for Biotechnology Information (NCBI) and its website (https://www.ncbi.nlm.NIh.gov). These sequences were analyzed using an online tool provided by iGeneTech (https:// mfeprimer3.Igene tech.com) to ensure that no primer dimers or hairpin structures were present. The primers and probes that passed the selection criteria were sent to Thermo Fisher Scientific (https://www.thermofisher.cn) for synthesis. To differentiate the amplification signals, the detection probes for LCR A-B, LCR B-D, and reference genes were labeled with FAM, VIC, and Cy5 fluorophores, respectively. The sequence information for primers and probes are presented in Additional file1: Supplement Table S1. Training set sample information are shown in Additional file1: Supplement Table S2. The information regarding positive samples for assessing probe effectiveness are shown in Additional file1: Supplement Table S3.

#### dPCR

The dPCR system used for detection was the QIAcuity One 5Plex System (Qiagen). In a 40 μL multiplex reaction, 27 μL DNA/cfDNA targets were added into 13 μL dPCR Probe Master Mix (Qiagen) containing eight primer pairs and the corresponding detection probes. The dPCR cycling conditions were as follows: 95 °C for 2 min and 40 cycles of 95 °C for 30 s, 58 °C for 30 s, and 72 °C for 30 s.

After amplification, the system automatically detected the fluorescence signal and calculated the copy number according to the Poisson distribution. The copy number of the target in each detection region (C_Tn_ (n = A-B, B-D)) or the reference gene (C_R_) was automatically calculated using the instrument’s software. The Z-score was calculated for each target detection region using the following equation:

$$Z_{\frac{Tn}{R}} = \frac{{\frac{{C_{Tn} }}{{C_{R} }} - {\text{NSMean}}\frac{{C_{Tn} }}{{C_{R} }}}}{{SD of NSMean\frac{{C_{Tn} }}{{C_{R} }}}}$$

where $Z_{\frac{Tn}{R}}$ is the Z-score of the copy number ratio between the target being tested and the reference gene $\left( {\frac{{C_{Tn} }}{{C_{R} }}} \right)$ from the same sample in the same dPCR assay. ${\text{NSMean}}\frac{{C_{Tn} }}{{C_{R} }}$ is the mean copy number ratio of normal samples that serves as a baseline for cut-off value determination, and SD is the standard deviation of ${\text{NSMean}}\frac{{C_{Tn} }}{{C_{R} }}$.

The risk of 22q11.2 deletion/duplication was determined according to the Z-score. If the absolute Z value was ≥ 3.67, there was a high risk of 22q11.2 deletion/duplication. Positive Z value indicated duplication in this region, while negative value indicated a deletion. If the absolute values of all Z values were < ​3.67, the risk of 22q11.2 deletion/duplication was low. If the absolute Z values were ≥ 3 and < ​3.67, they were in the gray zone and needed verification.

A total of 130 plasma samples were tested using dPCR, and all operators were unaware of the true status of the samples throughout the testing process for all samples and until results were obtained. Results of the dPCR were then compared with those of amniocentesis CMA.

### Statistical analysis

The data were analyzed using EmpowerStats software(X&Y solutions, inc.) and R (http://www.R-project.org) [28]. The sensitivity, specificity, positive predictive value (PPV), and negative predictive value (NPV) of the dPCR results were assessed. The receiver operating characteristic (ROC) curve was drawn and the area under curve (AUC) value was calculated.

## Results

### Preliminary assessment of probe effectiveness

Probes were set for the LCR A-B and LCR B-D regions of 22q11.2 deletion/duplication syndrome. Probe effectivity was verified by analyzing the DNA of four patients with differently sized 22q11.2 deletion/duplication as follows: Patient A: male, 28 yrs, 22q11.2 deletion 2.8 Mb (containing region LCRA-D); Patient B: female, 30 yrs, 22q11.2 deletion 1.4 Mb (containing region LCRA-B); Patient C: female, 23 yrs, 22q11.2 duplication 2.8 Mb (containing region LCRA- D); and Patient D: male, 27 yrs, 22q11.2 duplication (containing region LCRA-B) (Fig. 1). Results showed that the changes in Z values in each region of the four patients from A—D were consistent with their CNVs.

DNA with differently sized 22q11.2 deletion/duplication were detected by dPCR, indicating that the design of these probes and detection sites is reasonable and effective.

### Retrospective evaluation of cffDNA assay using multiplex dPCR

In the true positive group, we recruited 15 cases with fetal CNV in the 22q11.2 recurrent region, as confirmed by CMA. There were nine cases of 22q11.2 duplication, including two cases that involved the LCR A-B region, and the rest with the LCR A-D region. Deletion of the 22q11.2 region occurred in six cases, including two, one and three cases with the LCR A-B, LCR B-D, and LCR A-D regions, respectively (Fig. 1).

Eleven out of 15 positive cases with 22q11.2 deletion/duplication were detected through the dPCR assay, and accurate regional localization was achieved. The dPCR assay sensitivity for detecting fetal 22q11.2 deletion/duplication was 73.3% (Table 1). The clinical information, diagnostic results, and dPCR detection values of the 15 cases with 22q11.2 deletion /duplication in fetuses are shown in Table 2. Among the successfully detected 11 positive samples, seven, three and one contained LCR A-D, LCR A-B, and LCR B-D regions, respectively, in the 22q11.2 recurrent region. The dPCR assay accurately detected and indicated these abnormalities. Three cases (Patients 5, 6 and 14) were not successfully detected via dPCR, with two and one cases having LCR A-D and LCR A-B regions, respectively, with a size between 1.7–3.2 Mb. One 22q11.2 duplication sample with LCR A-D (Patient 10) was identified as a deletion (Table 3).

**Table 1:** The results of dPCR in retrospective study

CMA	n	dPCR	
		Positive	Negative
22q11.2 deletion /duplication	15	11	4*
Negative	115	4	111
total	130	15	115

*Contain one 22q11.2 duplication sample was identified as a deletion

**Table 2:** Detailed information of positive cases in this study

Positive Cases	Sample Information			CMA			dPCR			Fetal anomaly	Outcome	GA at delivery
	GA	MA	cfDNA (ng/ul)	Deletion/Duplication	Size (Mb)	Region	Z_A-B_	Z_B-D_	Final result			
	(WKS)	(YRS)										
Patient 01	20	23	0.240	duplication	2.8	LCRA-D	9.891	13.18	duplication(LCRA-D)	None	TOP	–
Patient 02	19	35	0.412	duplication	2.8	LCRA-D	12.568	15.193	duplication(LCRA-D)	None	TOP	–
Patient 03	21	31	0.368	duplication	2.9	LCRA-D	11.988	14.978	duplication(LCRA-D)	None	TOP	–
Patient 04	18 + 4	25	0.332	duplication	1.4	LCRA-B	12.765	− 0.293	duplication(LCRA-B)	None	TOP	–
Patient 05	18 + 3	26	0.320	duplication	2.8	LCRA-D	0.397	− 0.101	Negative	None	Live birth	Late preterm
Patient 06	18 + 3	35	0.128	deletion	3.2	LCRA-D	− 0.783	− 0.958	Negative	NT thickening	Live birth	Term
Patient 07	24 + 5	31	0.203	deletion	3.2	LCRA-D	− 18.274	− 21.151	deletion(LCRA-D)	Interrupted aortic arch, VSD	TOP	–
Patient 08	24 + 1	32	0.250	deletion	3.2	LCRA-D	− 3.716	− 4.635	deletion(LCRA-D)	Interrupted aortic arch	TOP	–
Patient 09	22 + 3	23	0.320	duplication	1.4	LCRA-B	12.481	− 0.661	duplication(LCRA-B)	None	Live birth	Term
Patient 10	26	21	0.284	duplication	2.8	LCRA-D	− 5.603	− 5.683	deletion(LCRA-D)	None	TOP	–
Patient 11	20 + 2	30	0.21	deletion	1.7	LCRA-B	− 13.633	− 0.8	deletion(LCRA-B)	None	TOP	–
Patient 12	23 + 5	21	0.348	duplication	3.2	LCRA-D	10.126	13.751	duplication(LCRA-D)	None	TOP	–
Patient 13	21	36	0.298	duplication	2.5	LCRA-D	15.868	17.86	duplication(LCRA-D)	None	TOP	–
Patient 14	19 + 5	34	0.466	deletion	1.7	LCRA-B	− 0.775	− 0.229	Negative	Interrupted aortic arch	TOP	–
Patient 15	18 + 5	26	0.284	deletion	0.8	LCRB-D	− 0.287	− 12.626	deletion(LCRB-D)	Interrupted aortic arch, VSD	TOP	–

Late preterm birth was defined as birth at 34 to 37 weeks’ gestation

*LCR* low-copy repeats, *GA* gestational age, *NT* nuchal translucency, *TOP* termination of pregnancy, *VSD* ventricular septal defect

**Table 3:** Detailed information of inconsistent samples detected by dPCR

Case	Sample Information			CMA			dPCR		
	GA	MA	cfDNA (ng/ul)	Deletion/Duplication	Size (Mb)	region	Z_A-B_	Z_B-D_	final result
	(WKRS)	(YRS)							
Patient 5	18 + 3	26	0.320	duplication	2.8	LCRA-D	0.397	− 0.101	Negative
Patient 6	18 + 3	35	0.128	deletion	3.2	LCRA-D	− 0.783	− 0.958	Negative
Patient 10	26	21	0.284	duplication	2.8	LCRA-D	− 5.603	− 5.683	deletion(LCRA-D)
Patient 14	19 + 5	34	0.466	deletion	1.7	LCRA-B	− 0.775	− 0.229	Negative
Negative case 23	23 + 5	31	0.415	Negative	–	–	5.202	2.1	duplication(LCRA-B)
Negative case 53	19	30	0.314	Negative	–	–	4.083	3.336	duplication(LCRA-B)
Negative case 76	21	33	0.438	Negative	–	–	− 4.771	− 5.122	deletion(LCRA-D)
Negative case 114	26 + 2	24	0.332	Negative	–	–	4.945	6.959	duplication(LCRA-D)

In the negative group, among the 115 pregnant women with normal fetuses, as confirmed by CMA detection, four cases showed high risk for 22q11.2 deletion/duplication in the dPCR assay (with one and three cases showing deletion and duplication, respectively), while 111 cases were considered to be normal. The false-positive rate and specificity were 3.5% and96.5%, respectively.

In this study, the positive and negative predictive values for the noninvasive detection of 22q11.21 deletion/duplication via dPCR assay were 73.3% and 96.5%, respectively.

### dPCR assay performance evaluation in plasma samples

**Fig. 2:**
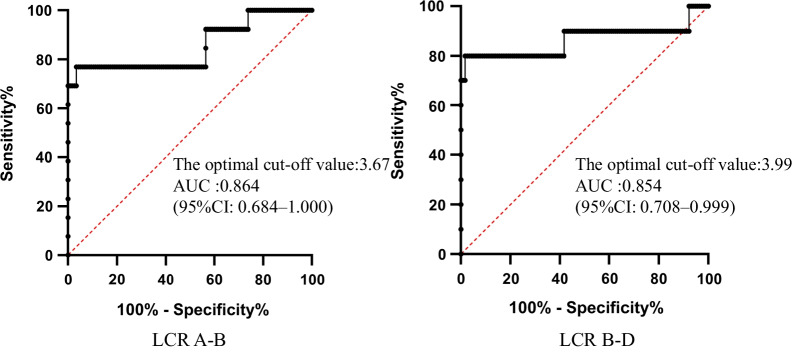
ROC curve analysis to assess the dPCR assay performance for identifying CNV in the LCR A-B and LCR B-D regions. The absolute value of a Z-score was used as a predictor of CNV for samples exceeding the cut-off value of 3.67 for the number of high risk results

A receiver operating characteristic (ROC) curve and the area under the ROC curve (AUC) are usually used to assess the performance of a diagnostic test [29]. Since one 22q11.2 duplication sample (Patient 10) was identified as a deletion, we plugged an ROC curve and calculated the AUC using the Z-scores of the 129 samples (Fig. 2). The AUC value was 0.854 (95% CI: 0.708–0.999) for the LCR A-B region and 0.864 (95% CI: 0.684–1.000) for the LCR B-D. This indicated that the dPCR assay was an efficient test method for screening fetal 22q11.2 deletion /duplication syndrome. Results of the ROC curve analysis supported that the Z-score cut-off value of the LCR A-B region was ± 3.67, suggesting that the Z-score cut-off value of the LCR B-D region was ± 3.99.

**Fig. 3:**
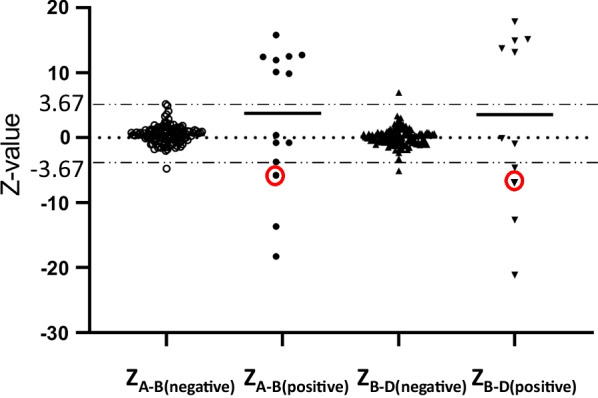
Z-score distributions for the LCR A-B and LCR B-D regions of 130 samples. The long horizontal dotted line indicates the cut-off value with a Z-score at 3.67. The short solid line in each data group represents the median Z-score value. The Z-scores of the 22q11.2 duplication sample identified as a deletion are circled in red

The positive detection rate for screening techniques should be as high as possible while maintaining the false-positive rate at an acceptable level. Our results verified that a Z-score cut-off value of 3.67 is appropriate for the available sample volume and dPCR system used. All the samples (130 samples) were sorted by Z-score (Fig. 3) with 96.5% of the negative samples being scattered between the Z-score cut-off lines of -3.67 and 3.67. Only four samples were beyond the two lines, with the absolute Z score of 4.08–6.96, which was close to the Z-score cut-off value. Most of the positive samples (73.3%) were scattered beyond the cut-off lines. The three cases which were scattered between the Z-score cut-off lines were not successfully screened.

## Discussion

The requirement for prenatal screening is justified by the high incidence of 22q11.2 deletion/duplication syndrome in prenatal fetuses resulting in the occurrence of a variety of severe abnormalities with long-term consequences, such as autism spectrum disorder and schizophrenia, and potential benefits of early neonatal therapy for hypocalcemia and immune deficiency in neonates [4, 7, 9]. In this study, we developed a dPCR assay for the noninvasive detection of 22q11.2 recurrent CNV in fetuses. Retrospective analysis preliminarily confirmed that the sensitivity, specificity, and PPV of this method were 73.3%, 96.5%, and 73.3%, respectively. Our study provided a method for noninvasive prenatal screening of 22q11.2 deletion/duplication syndrome. However, its effectivity needs to be further confirmed through prospective studies involving larger populations.

In approximately 85% of individuals affected by 22q11.2 deletion/duplication syndrome, copy number changes occur in the entire 2.5–3 Mb LCR A-D region (classical region), while others have smaller deletions/duplications within this region [27]. The LCR A-B region, containing many critical genes for determining the deletion/duplication syndrome, is associated with particular features and with similar clinical presentation as that of the classical region. Although the LCR B-D region has not been studied much, clinical features associated with these deletions/duplications, including heart defects and neuro developmental phenotypes, such as delay and autism spectrum disorders, overlap with those associated with classical deletion/duplication [27, 30]. In this study, target-specific sequences and probes were respectively set for two regions, the LCR A-B and LCR B-D. Due to the limited sample size of the retrospective study, the number of positive samples for the LCR A-B and LCR B-D were not equal, which led to different optimal Z-score cut-off values for both, as suggested by the ROC curve. Although the optimal Z-score cut-off value for LCR B-D differed after realignment, it did not affect the detection rate of positive samples. When the number of positive samples increased, the Z-score cutoff value was further optimized.

In the true positive group of this study, three cases (Patients 5, 6 and 14) were not detected by the dPCR assay, while one 22q11.2 duplication sample (Patient 10) was identified as a deletion (Table 3). By analyzing all test parameters, we found that the concentration of cfDNA extracted from Patient 6 was 0.128 ng/µL, which was much lower than that of other samples. The accuracy of dPCR is greatly affected when the cfDNA concentration is < 0.2 ng/µLin noninvasive prenatal screening or diagnosis, due to the high background of maternal DNA [19]. The data of Patients 5, 10 and 14 indicated that all parameters were normal. One possible cause for this may be the confined placental mosaicism (CPM). As cffDNA is mainly derived from cytotrophoblasts of chorionic villi in the placenta, it is not always representative of the fetus [31]. Unfortunately, we did not obtain placental tissue for verifying the CMP occurrence in these cases. Patient 10 was repeatedly identified as a deletion by the dPCR assay. We suspected that another possible cause to this conflicting result might be related to the microheterogeneities of the cfDNA target sequences present in this sample, which led to the unequal concentration of cfDNA fragments in the maternal plasma [32].

Given that the features of 22q11.2 deletion/duplication syndrome may not be apparent prenatally or during the clinical examination at birth, prenatal screening can prompt early diagnosis and intervention among high-risk pregnant women. In recent years, noninvasive prenatal screening based on NGS of cffDNA in maternal blood has introduced the potential for targeting any region of the genome, including an option to screen for sub-chromosomal CNVs [16, 33, 34]. However, the high false-positive rate of noninvasive prenatal testing leads to unnecessary invasive prenatal diagnosis increasing the difficulty of clinical prenatal consultation [35]. Some studies have achieved more accurate detection through increasing sequencing depth, customizing chips to add probes, and changing algorithms; however, these increase the cost involved [34, 36, 37]. Moreover, compared with dPCR, NGS is more expensive, more difficult to operate, and takes longer to provide results. The clinical application of NGS requires a more sophisticated interpretation of the results relying on bioinformatic databases.

In this study, a noninvasive assay based on dPCR was established to detect CNVs in the 22q11.2 recurrent region, and the screening effect was preliminarily evaluated on 130 clinical retrospective samples. Although the preliminary results suggested that the detection rate and specificity of the CNV in the 22q11.2 region are satisfactory, due to the limited sample size, there may be a deviation in the cut-off value, which requires more samples to be optimized. As a prospective study was not included in this study, the PPV of 22q11.2 deletion/duplication syndrome risk screening could not be obtained in the true sense.

## Conclusions

In this study, we developed a dPCR assay for noninvasive detection of 22q11.2 recurrent CNV in fetuses, which detected most affected cases, including smaller but relatively common nested deletions, with a low false-positive rate. Thus, we presented a potential, efficient, and simple dPCR assay for the noninvasive screening of 22q11.2 deletion/duplication syndrome in fetuses.

### Supplementary Information

**Additional file 1**. Supplementary tables.

## Data Availability

The datasets presented in this article are not readily available because Regulations on the management of human genetic resources in China. Requests to access the datasets should be directed to the corresponding author.

## Abbreviations

cffDNA: Cell-free fetal DNA
dPCR: Digital PCR
CNVs: Copy number variants
CMA: Chromosomal microarray
ACMG: American College of Medical Genetics and Genomics
LCR: Low-copy repeats
NCBI: National Center for Biotechnology Information
PPV: Positive predictive value
NPV: Negative predictive value
ROC: Receiver operating characteristic curve
AUC: Area under the curve
NGS: Next-generation sequencing
