# Supplementary material for: Preliminary study of noninvasive prenatal screening for 22q11.2 deletion/duplication syndrome using multiplex dPCR assay
Source: Orphanet J Rare Dis. 2023 Sep 8;18:278. doi: 10.1186/s13023-023-02903-2 (PMC10486099; doi:10.1186/s13023-023-02903-2)
Supplement: Supplementary file 1 — Additional file 1. Supplementary tables. [file 13023_2023_2903_MOESM1_ESM.docx]

Supplement Table1. Sequence information for primers and probes

| Region | Sequence Name | Sequence (5'-3') |
| --- | --- | --- |
| LCR22 A-B | primer1-F | ACTGGCTTAGTTGTCATTCCTCAT |
|  | primer1-R | CTGGGAAGCTCTGAGAAGGAAG |
|  | primer2-F | AAACCAGCAGAAAACGCTCG |
|  | primer2-R | GCCAGGTTTTGTAAACATTCAGGA |
|  | primer3-F | TTGACTGATACTGGCTGAAATGGA |
|  | primer3-R | GTGATCCTGGAAACCAACAGAATG |
|  |  |  |
| LCR22 B-D | primer1-F | GTCGATTAGGCAGGACTACAGG |
|  | primer1-R | GCCCCAACTTCATACCCTATACAA |
|  | primer2-F | AATCCTGGAATGAAGTGATGAGGG |
|  | primer2-R | TTCCTTCCTCTGGAATGTGACAC |
|  | primer3-F | GGGAGAGGTAAGGAATTAGTCACA |
|  | primer3-R | GACTCTGTCACTCAAGCCTTTCT |
|  |  |  |
| LCR22 A-B | probe1-P | TCTGGCCCTGTCCCCAGAGC |
|  | probe2-P | AGTTGCTGACCTGGGAAGTCGG |
|  | probe3-P | AGCTGGCCTCTGAATGTTGCAA |
|  |  |  |
| LCR22 B-D | probe4-P | GCCACCATGCCTGGCTGGT |
|  | probe5-P | GCCAGAGCTGAGCTGCAAGTGT |
|  | probe6-P | TCAGATGAGGGGCCTGGTGG |
|  |  |  |
| Reference Genes | primer1-F | CAAAGCATCACCTGTCCAGTTG |
|  | primer1-R | TGATGCCTTGAATGGACCGTTA |
|  | probe1-P | TCTCCTTTGATTTGTGATTTAAAGGT |
|  | primer2-F | GTTCGGCTTTCACCAGTCT |
|  | primer2-R | CTCCATAGCTCTCCCCACTC |
|  | probe2-P | CGCCCTGCCATGTGGAAGAT |

Supplement Table2. Training set sample information

| Case | Cy5  (copies/μL) | FAM  (copies/μL) | HEX  (copies/μL) | Result | |
| --- | --- | --- | --- | --- | --- |
|  |  |  |  | Z_A-B_ | Z_B-D_ |
| 1 | 139.6 | 230 | 242.1 | 0.50694823 | 2.131888 |
| 2 | 144.8 | 248.5 | 243 | 1.00641854 | 1.572296 |
| 3 | 139.7 | 238.9 | 240.2 | 0.96224134 | 1.983745 |
| 4 | 152 | 250.4 | 247.4 | 0.50552072 | 1.067789 |
| 5 | 146.2 | 253 | 249 | 1.11087645 | 1.821526 |
| 6 | 151.3 | 249.9 | 243 | 0.53695415 | 0.852682 |
| 7 | 148.4 | 260.1 | 254.2 | 1.27244444 | 1.919259 |
| 8 | 139.2 | 239.8 | 241.9 | 1.05404539 | 2.167288 |
| 9 | 140.9 | 236.7 | 232.3 | 0.74250285 | 1.27795 |
| 10 | 153.2 | 248.1 | 245.9 | 0.30224902 | 0.842808 |
| 11 | 139.6 | 237.7 | 237.9 | 0.90857048 | 1.83159 |
| 12 | 132.3 | 234.3 | 232.7 | 1.40554506 | 2.377833 |
| 13 | 230 | 374.9 | 365.3 | 0.37905501 | 0.674817 |
| 14 | 234.8 | 378.5 | 369.9 | 0.24806497 | 0.546282 |
| 15 | 227.5 | 367.5 | 382.1 | 0.27263514 | 1.586107 |
| 16 | 248.8 | 393.2 | 393.4 | 0.01779657 | 0.604238 |
| 17 | 240.2 | 368.2 | 376.9 | -0.3280432 | 0.483657 |
| 18 | 231 | 378.9 | 373.7 | 0.45375986 | 0.969147 |
| 19 | 234.3 | 375.9 | 380.1 | 0.19231285 | 1.014364 |
| 20 | 230.4 | 355.2 | 361 | -0.2641317 | 0.461012 |
| 21 | 232.6 | 347.4 | 353.6 | -0.614478 | -0.00446 |
| 22 | 209.7 | 341.2 | 346.8 | 0.35783941 | 1.328895 |
| 23 | 214 | 346.7 | 348 | 0.30692192 | 1.053183 |
| 24 | 243.4 | 312.6 | 382 | -2.1380696 | 0.486891 |
| 25 | 158.5 | 256.4 | 255.3 | 0.28924392 | 0.899044 |
| 26 | 151 | 234.7 | 242.8 | -0.1721101 | 0.871311 |
| 27 | 160.6 | 246.3 | 239.3 | -0.3226939 | -0.30558 |
| 28 | 63 | 102.8 | 93.2 | 0.39176849 | -0.41211 |
| 29 | 55.1 | 94.7 | 88.8 | 1.02486269 | 0.907925 |
| 30 | 89.7 | 156.2 | 146.3 | 1.18990784 | 1.101332 |
| 31 | 238.1 | 388.9 | 350.9 | 0.40342814 | -0.46815 |
| 32 | 212.4 | 332.4 | 300.1 | -0.094439 | -1.07551 |
| 33 | 96.1 | 143.8 | 147.2 | -0.5940407 | 0.110643 |
| 34 | 135.4 | 202.6 | 200.1 | -0.59441 | -0.42729 |
| 35 | 178 | 279.6 | 278.3 | -0.0520996 | 0.427503 |
| 36 | 104.8 | 162 | 160.3 | -0.2340243 | 0.089107 |
| 37 | 67.4 | 111.2 | 102.9 | 0.52360188 | 0.060421 |
| 38 | 115.1 | 190.9 | 177.5 | 0.58699444 | 0.214443 |
| 39 | 236.3 | 331.8 | 334.7 | -1.2654599 | -1.04038 |
| 40 | 240.6 | 372.2 | 352.8 | -0.225546 | -0.54217 |
| 41 | 296.9 | 447.4 | 436.6 | -0.5172462 | -0.5003 |
| 42 | 257.6 | 390.1 | 354.9 | -0.462937 | -1.42668 |
| 43 | 204.5 | 325 | 307.6 | 0.08228175 | -0.16465 |
| 44 | 172.4 | 259.4 | 261 | -0.5337335 | -0.06719 |
| 45 | 64.2 | 98 | 105.5 | -0.3747131 | 1.224202 |
| 46 | 371.3 | 502 | 550.9 | -1.6451158 | -0.36878 |
| 47 | 94.06 | 145.4 | 152.9 | -0.2338766 | 1.047093 |
| 48 | 100.7 | 153.4 | 158.9 | -0.3975992 | 0.571945 |
| 49 | 94.87 | 129.2 | 141.8 | -1.5733425 | -0.25927 |
| 50 | 84.3 | 141.2 | 130.2 | 0.70650024 | 0.237867 |
| 51 | 122.2 | 188.3 | 178.2 | -0.2695937 | -0.62271 |
| 52 | 72.09 | 115.9 | 112 | 0.2167723 | 0.328992 |
| 53 | 194.1 | 268.3 | 297.5 | -1.4247039 | 0.120398 |
| 54 | 66.96 | 116.1 | 104.6 | 1.13537685 | 0.413964 |
| 55 | 154.2 | 241.1 | 226.1 | -0.1047635 | -0.54274 |
| 56 | 170.8 | 265.1 | 253.4 | -0.1881066 | -0.36977 |
| 57 | 178.7 | 247.3 | 255 | -1.4130069 | -0.93505 |
| 58 | 187.9 | 275 | 266.6 | -0.8329678 | -1.01623 |
| 59 | 254.3 | 422.8 | 359 | 0.6164508 | -1.08731 |
| 60 | 37.76 | 62.56 | 57.27 | 0.57404734 | -0.03961 |
| 61 | 94.82 | 146 | 148.5 | -0.2780182 | 0.453875 |
| 62 | 94.09 | 128.6 | 143.1 | -1.53757 | 0.00231 |
| 63 | 542.9 | 993.2 | 814.2 | 1.83120941 | -0.2089 |
| 64 | 217.7 | 310.8 | 322.3 | -1.0943082 | -0.401 |
| 65 | 210.8 | 301.1 | 315.5 | -1.0890992 | -0.23929 |
| 66 | 163 | 239.5 | 250 | -0.7908763 | 0.130651 |
| 67 | 258.7 | 428.3 | 371.2 | 0.5653531 | -0.85626 |
| 68 | 187.4 | 284 | 306.4 | -0.4548433 | 1.141389 |
| 69 | 215.4 | 299.9 | 323.7 | -1.3517721 | -0.17834 |
| 70 | 249.5 | 359.1 | 392.7 | -1.0096564 | 0.531955 |
| 71 | 42.18 | 60.13 | 67.35 | -1.109567 | 0.759334 |
| 72 | 163.2 | 241.5 | 253.9 | -0.7147551 | 0.350414 |
| 73 | 152 | 224.6 | 235.6 | -0.7303942 | 0.292924 |
| 74 | 160.5 | 246.9 | 246.9 | -0.2885164 | 0.17632 |
| 75 | 60.08 | 95.55 | 90.03 | 0.09056387 | -0.22109 |
| 76 | 47.52 | 82.41 | 78.22 | 1.13789769 | 1.251567 |
| 77 | 18.06 | 29.43 | 29.43 | 0.37591023 | 1.087118 |
| 78 | 200.1 | 309.9 | 282.3 | -0.2127328 | -1.09653 |
| 79 | 192 | 282.2 | 265 | -0.7874794 | -1.40182 |
| 80 | 17.04 | 24.75 | 24.56 | -0.9136427 | -0.7919 |
| 81 | 144.8 | 211 | 215.8 | -0.8792926 | -0.30265 |
| 82 | 195.4 | 278 | 289.6 | -1.1302061 | -0.38493 |
| 83 | 91.68 | 140.6 | 137.5 | -0.3229036 | -0.20832 |
| 84 | 56.54 | 103.1 | 86.91 | 1.78790919 | 0.164583 |
| 85 | 92.73 | 145.7 | 141.4 | -0.0488829 | 0.041965 |
| 86 | 45.42 | 53.88 | 56.88 | -2.851964 | -2.6784 |
| 87 | 232.9 | 341.2 | 345.1 | -0.8223224 | -0.38828 |
| 88 | 273 | 402.3 | 407.5 | -0.7595576 | -0.27927 |
| 89 | 182.9 | 280.8 | 258.1 | -0.3107433 | -1.09295 |
| 90 | 170.4 | 251.7 | 248.3 | -0.7341725 | -0.63374 |
| 91 | 55.41 | 82.9 | 90.06 | -0.5957746 | 1.0449 |
| 92 | 291 | 428.3 | 427.9 | -0.7727009 | -0.50112 |
| 93 | 157 | 253 | 243.9 | 0.24409478 | 0.32789 |
| 94 | 210.2 | 261.3 | 303.8 | -2.4380898 | -0.75222 |
| 95 | 191.8 | 301 | 269.9 | -0.0626099 | -1.13246 |
| 96 | 256.6 | 417 | 373.4 | 0.34335762 | -0.65347 |
| 97 | 222.2 | 335.9 | 329.9 | -0.4823213 | -0.35887 |
| 98 | 242.9 | 362.6 | 368.1 | -0.6199811 | -0.05205 |
| 99 | 262.4 | 439.4 | 395.9 | 0.70338629 | -0.11865 |
| 100 | 166.5 | 284.3 | 229.3 | 0.94341502 | -1.43207 |
| 101 | 170.2 | 298.1 | 249.3 | 1.26351343 | -0.55801 |
| 102 | 265.6 | 468 | 411.9 | 1.34054535 | 0.301191 |
| 103 | 84.02 | 155.9 | 127.9 | 2.02107867 | 0.016008 |
| 104 | 101.9 | 174.2 | 164.1 | 0.95806242 | 0.895819 |
| 105 | 83.14 | 173.9 | 124.6 | 3.74051374 | -0.21935 |
| 106 | 238.7 | 418.9 | 350.6 | 1.2886606 | -0.51767 |
| 107 | 94.85 | 164.9 | 140.7 | 1.16933391 | -0.37188 |
| 108 | 195.5 | 329.5 | 262 | 0.78260247 | -1.80162 |
| 109 | 263.2 | 403.2 | 387.5 | -0.3351379 | -0.48298 |
| 110 | 204.7 | 329.1 | 298.6 | 0.21681622 | -0.61817 |
| 111 | 100 | 198.3 | 139.8 | 2.94937486 | -1.22424 |
| 112 | 80.91 | 141.1 | 128.1 | 1.20849642 | 0.624709 |
| 113 | 27.81 | 41.58 | 44.01 | -0.6028681 | 0.617559 |
| 114 | 85.59 | 138.5 | 123.5 | 0.29298711 | -0.77582 |
| 115 | 85.02 | 127.1 | 125.8 | -0.6043482 | -0.40924 |
| 116 | 290.1 | 558.2 | 405.8 | 2.5209694 | -1.21597 |
| 117 | 787.6 | 1245.1 | 1138.8 | 0.02138458 | -0.74601 |
| 118 | 294.7 | 461 | 415.7 | -0.0993108 | -1.0986 |
| 119 | 372.3 | 579.5 | 526.7 | -0.1558306 | -1.05735 |
| 120 | 108.9 | 178.3 | 166.1 | 0.43207721 | 0.045911 |
| 121 | 72.94 | 106.7 | 107.4 | -0.8380521 | -0.4812 |
| 122 | 105.1 | 169.7 | 145.1 | 0.26730589 | -1.39801 |
| 123 | 31.06 | 43.74 | 47.32 | -1.2356439 | 0.028448 |
| 124 | 172.5 | 248.9 | 247.5 | -0.9832979 | -0.8571 |
| 125 | 97.92 | 164.3 | 146.3 | 0.7278345 | -0.26526 |
| 126 | 61.39 | 96.42 | 88.4 | -0.053355 | -0.80528 |
| 127 | 95.98 | 152 | 145.1 | 0.04166058 | -0.08863 |
| 128 | 218 | 309.1 | 300.1 | -1.1653948 | -1.43778 |
| 129 | 229 | 332.6 | 309.5 | -0.9141017 | -1.68808 |
| 130 | 50.9 | 86.55 | 88.39 | 0.89161121 | 2.15486 |
| 131 | 92.47 | 149.2 | 142.7 | 0.25888541 | 0.225083 |

Supplement Table3. The information of positive sample for assessment of probe effectiveness

| Case | Cy5  (copies/μL) | FAM  (copies/μL) | HEX  (copies/μL) | Result | |
| --- | --- | --- | --- | --- | --- |
|  |  |  |  | Z_A-B_ | Z_B-D_ |
| Patient A | 236.4 | 179.6 | 174.1 | -5.9576989 | -7.82723665 |
| Patient B | 221.3 | 200.4 | 335.2 | -4.8958671 | -0.05956 |
| Patient C | 163.6 | 354.1 | 344.5 | 4.2703986 | 5.83999519 |
| Patient D | 182.1 | 390.4 | 259.2 | 4.12077805 | -0.97077 |
